# Post-transcriptional dysregulation in autism, schizophrenia, and bipolar disorder

**DOI:** 10.7555/JBR.38.20240114

**Published:** 2024-05-29

**Authors:** Yuanyuan Wang, Yitong Yan, Bin Zhou, Mingyan Lin

**Affiliations:** 1 State Key Laboratory of Reproductive Medicine and Offspring Health, Nanjing Medical University, Nanjing, Jiangsu 211166, China; 2 Department of Neurobiology, School of Basic Medical Sciences, Nanjing Medical University, Nanjing, Jiangsu 211166, China; 3 Department of Cardiology, the First Affiliated Hospital of Nanjing Medical University, Nanjing, Jiangsu 210029, China

**Keywords:** post-transcriptional gene regulation, psychiatric disorders, RNA-binding protein, ELAVL3

## Abstract

The alteration of gene expression is not restricted to transcriptional regulation but includes a variety of post-transcriptional mechanisms; however, the role of the latter in many diseases remains relatively unknown. By using an RNA-Seq dataset of 1510 brain samples from individuals with autism spectrum disorder (ASD), bipolar disorder (BD), schizophrenia (SCZ), and controls, we assessed the contribution of post-transcriptional dysregulation and identified top perturbators accountable for transcriptomic alterations in neuropsychiatric disorders. Approximately 30% of the expression variability was attributed to post-transcriptional dysregulation. Interestingly, mature mRNA levels tended to be post-transcriptionally downregulated in SCZ and BD, leading to the inhibition of neurogenesis and neural differentiation, while they were upregulated in ASD, resulting in enhanced activity of apoptosis. These findings imply contrasting pathologies involving RNA metabolism across neuropsychiatric disorders. An RNA-binding protein, ELAVL3, was predicted to be significantly involved in the disruption of post-transcriptional regulation in all three disorders. To validate this, we knocked down its expression in cerebral organoids. Not only did the differentially expressed genes in *ELAVL3* knockdown cover a considerable proportion of predicted targets in the three disorders, but we also found that neurogenesis was significantly affected, given the diminished proliferation and consequently reduced size of the organoids. The present study extends the current understanding of the link between post-transcriptional regulation and neuropsychiatric disorders and provides new potential therapeutic targets for early intervention.

## Introduction

Genetic factors are believed to play an important role in the pathogenesis of neuropsychiatric disorders, such as schizophrenia (SCZ), bipolar disorder (BD), depression, and autism spectrum disorder (ASD) ^[ 1] ^. Thanks to advances in sequencing technology, recent studies have revealed that the majority of risk loci for neuropsychiatric disorders lie in regulatory regions ( *i.e.*, non-coding gene regions) rather than coding regions ^[ 2] ^, suggesting that the dysregulation of gene expression and alternative splicing is more relevant to the development of neuropsychiatric disorders than the malfunction of genes. Gene expression is jointly regulated by transcriptional (TGR) and post-transcriptional (PTGR) mechanisms. The former has been the focus of decades of research, leading to an extensive understanding of the roles of many transcription factors and epigenetic patterns in neuropsychiatric disorders, including our previous studies on the SCZ risk genes *ZNF804A* and *TCF4* ^[ 3] ^, 22q11.2 microdeletion, and ASD-related gene *CHD*8 ^[ 4] ^. PTGR contributes to almost every aspect of RNA metabolism, such as splicing, maturation, transportation, and degradation ^[ 5] ^. mRNAs are primarily regulated by microRNAs (miRNAs) and RNA-binding proteins (RBPs). Despite the long debate regarding which plays a more important role in PTGR, emerging evidence suggests that RBPs, given their involvement in many aspects of RNA metabolism, have a much larger influence than miRNAs, which mainly participate in mRNA degradation ^[ 6] ^. However, our current knowledge about the association between RBPs and neuropsychiatric disorders is limited to a few genes, such as *FMR1*, *DISC1*, and the members of the *RBFOX* family. Recent studies have significantly expanded the pool of RBPs, the number of which is estimated to be at least 800 ^[ 7] ^. Therefore, a transcriptome-wide assessment of PTGR dysregulation and the identification of potential risk RBPs are highly valuable in deciphering complex neuropsychiatric disorders.

Several studies have shown that alterations in TGR and PTGR may be evaluated simultaneously using RNA-seq data. The rationale is that the number of reads mapped to intronic regions represents the expression levels of pre-mRNAs, which are mainly transcriptionally regulated; the number of reads mapped to exonic regions represents the expression levels of mature mRNAs, which are both transcriptionally and post-transcriptionally regulated ^[ 8– 9] ^. Hence, subtraction of changes in exonic levels (Δexon) from changes in intronic levels (Δintron) may reflect the alteration in PTGR across conditions, though many technical and biological factors should be taken into account to avoid biases that may overestimate the change in PTGR ^[ 10] ^.

Following the idea above, we developed an unbiased algorithm to assess PTGR dysregulation in three common psychiatric disorders (ASD, SCZ, and BD) based on a large public dataset of 1510 brain transcriptome data from the PsychENCODE Consortium ^[ 11] ^, including 80 ASD samples, 428 SCZ samples, and 188 BD samples. We found PTGR dysregulation contributed significantly to expression changes in diseases and identified the most likely responsible RBPs. To validate our algorithm, we knocked down the most relevant RBP, ELVAL3, in cerebral organoids and examined its role in neurodevelopment. In summary, our present study provided an unbiased and reliable method to assess PTGR dynamics, highlighted the involvement of PTGR dysregulation underlying neuropsychiatric disorders, and listed several potential targets for further research from the post-transcriptional perspective.

## Materials and methods

### Ethics and data collection

The Institutional Review Board of Nanjing Medical University approved the study (Approval No. [2020]525).

For the present study, all processed bulk RNA-seq alignment (.bam) files were obtained from the PsychENCODE Consortium ( https://www.synapse.org/#!Synapse:syn6039873). Clinical data of Bulk RNA-Seq samples are available at https://www.synapse.org/#!Synapse:syn4587614. We used hg19 as the reference genome for all files. Of the 2160 samples in the PsychENCODE Consortium, 1510 are rRNA-depleted sequencing samples with sufficient intronic reads (control, *n* = 814; SCZ, *n* = 428; BD, *n* = 188; and ASD, *n* = 80). We analyzed the 1510 samples subsequently.

### Annotation of exon and intron reads

We extracted intron coordinates of genes from Gencode (v19) to generate an intron gene transfer format (GTF) annotation file. We extracted the coordinates of exons and untranslated regions within any isoform of a gene from Gencode.v19.gtf as the composite exon GTF annotation file. We used featureCounts ^[ 12]^ (version 2.0.3) to separately quantify the abundance of exons and introns at the gene level.

### Covariate selection

We calculated the transcripts per million (TPM) of each gene using exon abundance. For subsequent analysis, we only retained genes with TPM > 1 in at least 50% of the samples.

Missing values in clinical data were imputed using the R package "missMDA" (version 1.19) ^[ 13] ^.

To determine which covariates would affect the evaluation of gene post-transcriptional regulation, we performed multivariate adaptive regression using the R package "earth" (version 5.3.3) ^[ 14]^ on the intronic and exonic counts. We included a superset of potential covariates available for all samples, including study, tissue, libraryPrep, strand specificity, platform, individual ID source, diagnosis, sex, ethnicity, PMI, RIN, ageDeath, and all 40 sequencing principal components, along with squared terms for continuous variables. The gene expression data and covariates were normalized using the R package "limma" ^[ 15]^ and served as inputs for the "earth" model analysis. As the model fits a maximum of 1000 genes simultaneously, we performed 1000 permutations, randomly sampling 1000 genes at a time. Finally, we selected a set of covariates present in both exons and introns, including study, diagnosis, sex, ethnicity, PMI, ageDeath, PC (1-3), PC (5-11), PC (13-18), PC20, PC22, PC23, PC (25-27), PC29, PC31, PC (33-36), PC39, RINS2, ageDeathS2, PC1S2, PC2S2, PC4S2, PC6S2, PC9S2, PC16S2, PC17S2, and PC22S2.

### Exonic/Intronic counts correction

Exonic/intronic counts were normalized for library size using the trimmed mean of *M*-values (TMM) normalization in the R package "edgeR" (version 3.40.2) ^[ 16]^ and were transformed to log _2_(counts per million [CPM]). We used the R package "nlme" (version 3.1-162) ^[ 17]^ to correct exonic and intronic counts based on the linear mixed-effects model. We treated the log _2_CPM counts of exonic/intronic data as the dependent variable in the model, with the covariates and disease groups specified in the previous section as fixed effects. In addition, each unique subject was treated as a random effect in the model. All covariates except diagnosis and subject were regressed from our exonic/intronic count dataset. Exonic and intronic counts were analyzed separately.

### New PTGR estimation pipeline

Reads in the intron region reflect the expression levels of pre-mRNA, and reads in the exon region reflect the expression levels of mature mRNA ^[ 8] ^. We subtracted the median exon count of each gene from the exon count of all samples to obtain the change in exon count (Δexon). The analysis process for the change in intron count (Δintron) followed the same approach.

For each set (control, SCZ, ASD, and BD), we used robust linear regression to model the ratio of Δexon to Δintron for each gene. The control group ratio was recorded as *slope* A. We used the same method to estimate the ratio of ∆exon and ∆intron in the disease groups. The disease group ratio was recorded as *slope* B. If B > A, this indicates that mature mRNA levels are post-transcriptionally up-regulated in the disease group (∆PTGR > 0). Positive and negative ∆PTGR values correspond to post-transcriptionally upregulated and downregulated mature mRNA levels, respectively. The test shown below was used to quantify the statistical significance of ∆PTGR:

1 \begin{equation*}\begin{split} & t=\frac{slope_{\mathrm{Disease}}-slope_{\mathrm{Ctrl}}}{\sqrt{S E_{\mathrm{Disease}}^2+S E_{\mathrm{Ctrl}}^2}}, \\ & df=\mathrm{size}_{\mathrm{Disease}}+\mathrm{size}_{\mathrm{Ctrl}}. \end{split}\end{equation*}

Here, the *slope* represents the robust linear regression slope value, and *SE* represents the standard error of the *slope*. The *df* is the sum of samples in the control (Ctrl) and disease groups.

For each gene, the effect of PTGR versus TGR on gene expression was estimated according to the following formula:

2 $ ratio=\left|\frac{slope_{\mathrm{Disease}}}{slope_{\mathrm{Ctrl}}}-1\right|. $

This ratio represents the relative contribution of PTGR dysregulation of each gene to changes in gene expression levels, compared with TGR dysregulation.

The average ∆PTGR of each gene was obtained from the above analysis. To calculate the ∆PTGR of each gene in each individual ( *∆PTGR*_in_) with the disease, we used the following formula:

3 $ \Delta PTG{R}_{in}=\Delta exon_{\mathrm{Disease}}-\Delta intron_{\mathrm{Disease}}\times slope_{\mathrm{Ctrl}}. $

### Post-transcriptional perturbation dataset analysis

We used RNA-seq data of schizophrenia patient-derived neural progenitor cells (GEO accession GSE80170) with microRNA-mediated post-transcriptional perturbation ^[ 18]^ to evaluate the performance of the PTGR algorithm. The data include control samples, samples with the miR-9 knockdown (KD), and samples with the miR-9 overexpression. We used STAR ^[ 19]^ with default parameters to align reads to the hg19 reference genome. We used custom intronic GTF and composite exonic GTF for read annotation, and gene-level abundance was quantified for intronic and exonic regions separately using featureCounts. We identified genes inferred to be up-PTGR or down-PTGR in the knockdown/overexpression group. Because miRNA targets the miRNA seed in the 3′ UTR to exert regulatory effects, we examined whether genes that were up-PTGR or down-PTGR were enriched for miR-9 RNA seed sites in their 3′ UTRs to evaluate the performance of the PTGR algorithm.

### Integrated PsychENCODE transcription regulatory results

The results of the differentially expressed genes (DER-13_Disorder_DEX_Genes.csv) of three neuropsychiatric disorders were retrieved from the PsychENCODE database ( http://resource.psychencode.org).

### Identification of key RBPs

We first downloaded the position frequency matrices (PFMs) of human RBPs from the beRBP database ( http://bioinfo.vanderbilt.edu/beRBP). We used the MoSBAT ^[ 20]^ (version 1.0.0) to assess the similarities of all PFMs and cluster the PFMs to generate the non-redundant RBP binding PFM set. Then, we used MoSBAT to perform RBP PFM scanning in the 3′ UTR sequence of the differentially post-transcriptionally regulated genes (DPRGs) to obtain vectors for RBP binding on DPRGs. To reduce the alternative splicing confounding effects, we only included genes with the same 3′ UTR start coordinates for all isoforms. We used the RNA stability measures of DPRGs as the response variable in linear regression, with the RBP binding vector of DPRGs as predictor variables. RBPs and miRNAs significantly associated with disease-specific changes in post-transcriptional regulation of mature mRNA levels were identified based on the regression coefficients at *P-*value < 0.05. If the gene's 3′ UTR sequence contains an RBP binding site, the gene is predicted to be the binding target of the RBP (RBP-bound gene).

### Development of human cerebral organoids

The wild-type human induced pluripotent stem cell lines (iPSC) (NC3-1, passage 16; ihtc-03, passage 20) were provided by Dr. Yan Liu's laboratory (Nanjing Medical University, China). All stem cell lines were cultured with essential eight (E8) medium (Life Technologies, Carlsbad, CA, USA) at 37 ℃ and 5% CO _2_, and the E8 medium was changed daily.

After culturing iPSC clones in E8 medium for seven days, the iPSCs were dissociated using ethylenediaminetetraacetic acid (Lonza, Houston, TX, USA) at 37 ℃ for 1 min. Next, we seeded the iPSCs in a 6-well plate at a density of approximately 1 × 10 ^5^ cells per well. We used dispase (Life Technologies) to detach the iPSCs. Then, we cultured the detached iPSCs in the neural induction medium, composed of 490 mL DMEM/F12 medium (Life Technologies), 5 mL of N2 supplement (Gibco, Grand Island, NY, USA), and 5 mL of minimum essential medium non-essential amino acids (Gibco) to form embryoid bodies (EB). Half of the neural induction medium was changed daily from day 1 to day 6. On day 7, we resuspended the EB in Matrigel (Corning, Corning, NY, USA) and cultured it in the differentiation medium, which was changed every five days.

### Genome editing

Genome editing of cerebral organoids was performed by Promoter Biotechnology (Jiangsu, China). We used CRISPR/Cas9 genome editing technology to generate the *ELAVL3* KD NC3-1 and ihtc-03 iPSC lines. The guide RNA sequences of *ELAVL3* for the targeting site were as follows: forward, 5′-CTTGTCCCGAACCAACTTGC-3′; reverse, 5′-TTTGTACCAAGGAGTGGCCC-3′. We used the TRIzol kit (Thermo Fisher Scientific, Waltham, MA, USA) to extract total RNA and then reverse transcribed it into cDNA using the SuperScript Ⅲ First-Strand Synthesis System (Thermo Fisher Scientific). We conducted qPCR on cDNA using the AceQ Universal U+ Probe Master Mix V2 kit (Vazyme, Nanjing, China). The primers used for qPCR were as follows: *ELAVL3* forward primer, 5′-TCGAGTCCTGCAAGTTGGTTC-3′; reverse primer, 5′-TGCATCATTGGGGTCAGAATAGT-3′.

### Immunostaining

We used 4% paraformaldehyde (Beyotime, Shanghai, China) to fix organoids in the Eppendorf tube for 2 h and used phosphate-buffered saline (Beyotime) to wash them three times. Next, we used PBS with 20% sucrose (Beyotime) to submerge the organoids at 4 ℃ overnight. When the organoids sank to the bottom of the tube, we replaced the soaking solution with 30% sucrose in PBS at 4 ℃. We embedded the organoids in OCT compound (SAKURA, Tokyo, Japan) and cryosectioned them at 10 μm to generate tissue sections. We first washed the organoid sections with PBS three times and then used 1% Triton (Biolink, Shanghai, China) and 5% donkey serum (Millipore, Darmstadt, Hesse, Germany) in PBS to block and permeabilize the organoid sections. Subsequently, we used the primary antibody diluted in 0.2% Triton and 5% donkey serum to incubate sections at 4 ℃ overnight. Then, we used the secondary antibody diluted in 5% donkey serum to incubate sections at 20 ℃ for 1 h. We used PBS to perform three 10-min washes. Finally, we fixed the mounted coverslip and used the Eclipse 80i fluorescence microscope (Nikon, Japan) for imaging. Antibodies used in this study included Ki67 (Rabbit IgG, dilution 1∶200; Cat. #18-0191, ZYMED, Carlsbad, CA, USA) and MAP2 (Mouse IgG, dilution 1∶1000, Cat. #M1406, Sigma, St. Louis, MO, USA).

### Bulk RNA-Seq data analysis

Total RNA from day-30 *ELAVL3*-KD and control cerebral organoids from both NC3-1 and ihtc-03 iPSC lines was extracted, and library construction was conducted by Annoroad Gene Technology (Beijing, China). Sequencing was performed with the DNBSEQ-T7 platform (BGI, Shenzhen, China). We used HISAT2 software (version 2.1.0) ^[ 21]^ to align reads to the hg19 reference genome. Gene-level abundance was quantified using featureCounts. Differentially expressed genes between the *ELAVL3*-KD and control cerebral organoids were identified using the R package "DESeq2" (version 1.38.3) ^[ 22] ^. Genes with |log _2_(fold change)| > 0 and adjusted *P*-value < 0.05 were treated as significantly differentially expressed.

We used STAR ^[ 19]^ to align reads to the hg19 reference genome. We used custom intronic GTF and composite exonic GTF for read annotation, and gene-level abundance was quantified for intronic and exonic regions separately using featureCounts. We used the R package "edgeR" (version 3.40.2) ^[ 16]^ to normalize exonic/intronic counts and transform exonic/intronic counts as log _2_CPM. Subtraction of changes in exonic levels (Δexon) by changes in intronic levels (Δintron) across *ELAVL3* KD and control conditions represented the change of post-transcriptional regulation of mature mRNA levels in *ELAVL3* KD organoids.

### Developmental expression data

The expression data of the developing human brain were obtained from the BrainSpan database ( http://www.brainspan.org).

### Enrichment analysis

We used the R package "clusterProfiler" (version 4.6.0) ^[ 23]^ to perform Gene Ontology (GO) enrichment analysis. The GO interactome network was generated by Metascape (version 3.5) ^[ 24] ^.

To determine whether differentially expressed genes (DEGs) in *ELAVL*-KD cerebral organoids were significantly enriched with genes associated with psychiatric disorders, we overlapped the DEGs with the susceptibility gene lists of the autism DB database, SFAIR autism database, BipEX database, and data from Fromer *et al* ^[ 25] ^, Gulsuner *et al* ^[ 26] ^, and Li *et al* ^[ 27] ^. Then, we used Fisher's exact one-sided test to perform susceptibility gene enrichment analysis.

We used MEME (version 5.5.0) ^[ 28]^ with default parameters to identify the motif of 3′ UTR sequence.

### Statistical analysis

Experimental statistical data were reported as mean ± standard error of the mean. Student's *t*-test was used for statistical analysis between two groups, and ANOVA was used for statistical analysis of multiple groups. Other statistical tests are listed in the legend or methods section of each figure.

## Results

### An unbiased pipeline for assessing the contribution of PTGR dysregulation to gene expression

**Figure 1:**
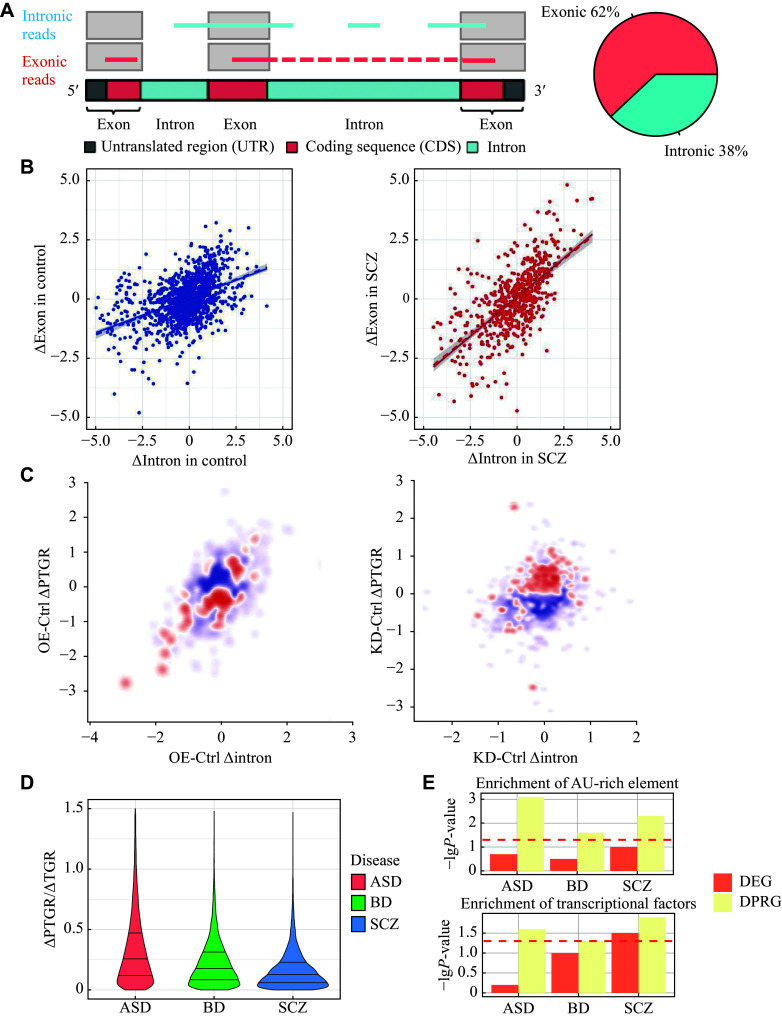
An unbiased pipeline for assessing the contribution of post-transcriptional gene regulation (PTGR) dysregulation to gene expression. A: Schematic diagram of the theoretical basis for the PTGR estimation algorithm (left). The fraction of exonic and intronic reads in the PsychENCODE bulk RNA-Seq dataset is shown in the pie chart (right). The intronic reads represent the abundance of pre-mRNAs, and the exonic reads represent the abundance of mature mRNAs. B: Schematic diagrams of the PTGR estimation method. Scatter plots comparing the PTGR in control (left) and SCZ (right) groups. The y-axis represents the changes in exonic reads. The x-axis represents the changes in intronic reads. Each dot represents a sample, and the solid line represents the fitted line. C: The scatter plot shows the miR-9 seed sequence enrichment in genes under miR-9 overexpression (OE; left) and miR-9 knockdown (KD; right) conditions. The y-axis represents the changes in exonic reads. The x-axis represents the changes in intronic reads. Each dot represents a gene, and the color represents the degree of enrichment of genes with miR-9 seed sequence in the 3′ UTR, with red representing enrichment and blue representing depletion. D: The violin plot shows the change ratio of PTGR changes (disease versus control) to transcriptional gene regulation (TGR) changes (disease versus control) using the formula (2) in the ***Materials and methods*** section. The ratio represents the relative contribution of PTGR dysregulation to changes in gene expression levels compared with that of TGR dysregulation of each gene. The average ratio of all genes and all diseases was 0.30 (30%). The three lines within the violin plot represent the 75%, median, and 25% percentiles from top to bottom. ASD, BD, and SCZ are displayed in red, green, and blue, respectively. E: The bar plot shows the degree of enrichment of regulatory elements AREs (top) and transcription factors (bottom) in DPRGs and DEGs across the three diseases using Fisher's exact test. Abbreviations: Ctrl, control; ASD, autism spectrum disorder; BD, bipolar disorder; SCZ, schizophrenia; ARE, adenylate-uridylate (AU)-rich element; DPRG, differentially post-transcriptionally regulated gene; DEG, differentially expressed gene.

To obtain an unbiased estimate of gene expression changes caused by post-transcriptional dysregulation, we developed a computational analysis pipeline for RNA-seq data as follows. Changes in intronic read abundance (Δintron) across different conditions reflect transcriptional-level alterations, while changes in exonic read abundance (Δexon) reflect both transcriptional and post-transcriptional regulation (see the *Materials and methods* section; ***Fig. 1A***). We first quantified the abundance of exonic and intronic reads in PsychENCODE RNA-seq datasets. As expected, we found that approximately 38% of reads were intronic, and 62% were exonic ( ***Fig. 1A***). This observation is consistent with previous studies ^[ 8– 9] ^, suggesting that there are sufficient reads from pre-mRNA for our analytical purposes. We then modeled the difference between Δexon and Δintron (∆PTGR) for each gene in each group using robust linear regression to remove biases from confounding factors ( *Materials and methods* and ***Fig. 1B***). If the regression slope in the disease group is larger than that in the control group, this indicates that mature mRNA levels are post-transcriptionally up-regulated in the disease group (∆PTGR > 0).

To evaluate the performance of our pipeline, we estimated ΔPTGR using RNA-seq data from SCZ patient-derived neural progenitor cells with microRNA-9 (miR-9)-mediated post-transcriptional perturbations ^[ 18] ^. miRNA degrades its targets by binding their 3′ UTR with the seed region. As expected, our results showed that genes downregulated at the PTGR level in the miR-9 overexpression condition and upregulated at the PTGR level in the miR-9 knockdown condition were significantly enriched for miR-9 direct targets ( ***Fig. 1C***).

To assess the relative contribution of TGR and PTGR dysregulation to expression changes across the three common psychiatric disorders, we next applied our pipeline to the 1510 brain transcriptome samples from the PsychENCODE Consortium. Our analysis revealed that the relative contribution of PTGR dysregulation to the change in gene expression levels was, on average, approximately 30% of that of TGR dysregulation ( ***Fig. 1D***), although a small proportion of genes had expression altered primarily in a post-transcriptional manner. Moreover, we found that transcription factors were significantly overrepresented in DPRGs compared with DEGs ( ***Fig. 1E***), indicating that PTGR dysregulation may profoundly affect gene expression through many upstream regulators. Our work uncovered, for the first time, how important PTGR dysregulation could be to the pathogenesis of common neuropsychiatric disorders in terms of gene expression.

### Transcriptome-wide targets of PTGR dysregulation in neuropsychiatric disorders

**Figure 2:**
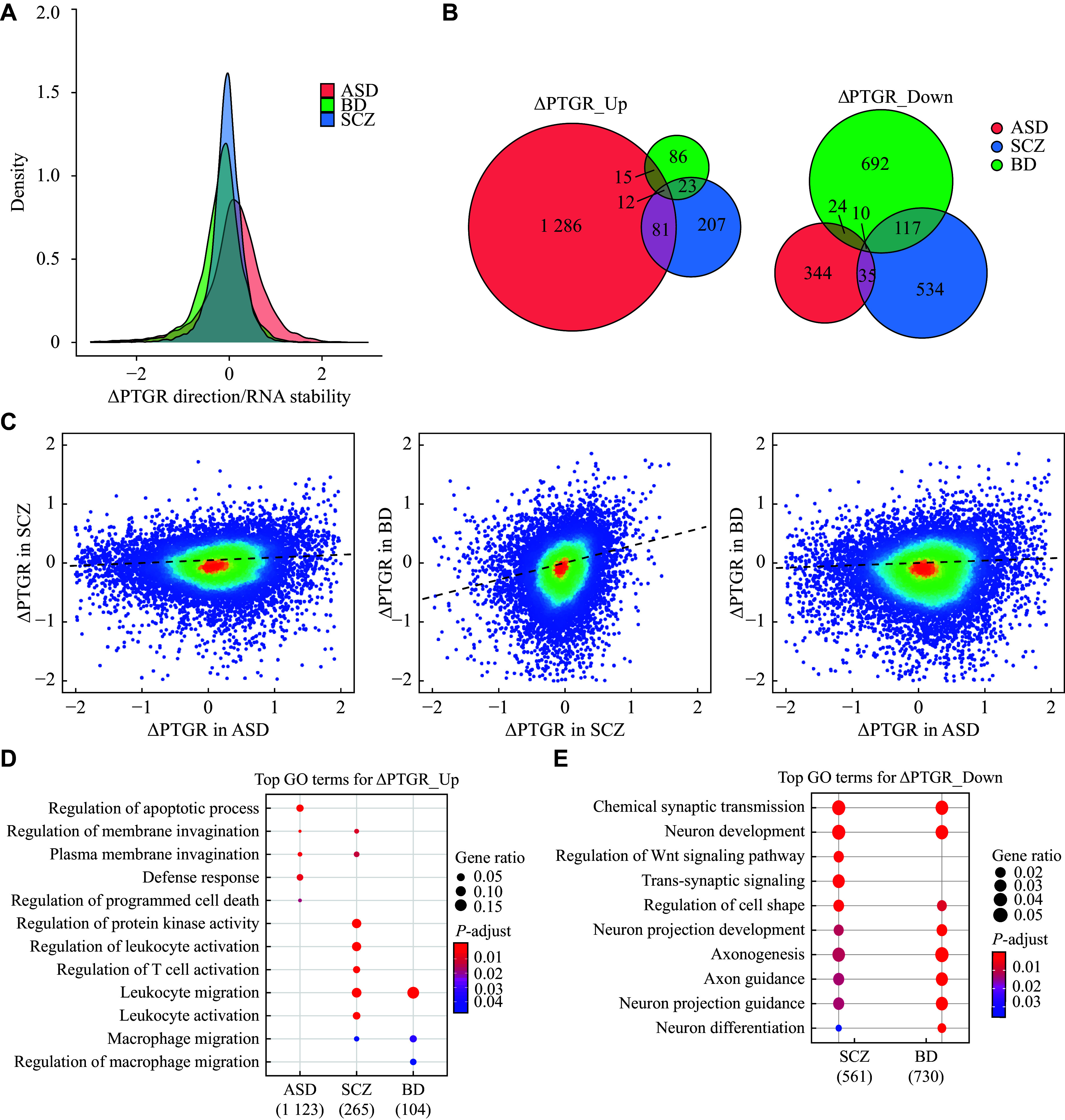
Transcriptome-wide targets of post-transcriptional gene regulation (PTGR) dysregulation in neuropsychiatric disorders. A: The density plot shows the distribution of ∆PTGR of the expressed genes across the three diseases. The ∆PTGR > 0 represents an increase in RNA stability (disease versus control), whereas ∆PTGR < 0 represents a decrease in RNA stability (disease versus control). B: The Venn diagram shows the overlap of significantly differentially post-transcriptionally regulated (DPRG) genes (FDR < 0.05). Genes for ASD, BD, and SCZ are displayed in red, green, and blue, respectively. C: The scatter plot shows the correlation of ∆PTGR between diseases. Each dot represents a gene. The color represents the localized density of genes. Red represents high gene density, and blue represents low gene density. The dotted line represents the fitted line. D and E: The gene ontology functional enrichment results of upregulated (D) and downregulated (E) DPRGs across diseases. Numbers in parentheses represent the count of genes in each disorder. Abbreviations: ASD, autism spectrum disorder; BD, bipolar disorder; SCZ, schizophrenia; FDR, false discovery rate.

We first assessed post-transcriptional alteration genome-wide in ASD, SCZ, and BD, compared with the control. The results showed that ASD was not only more vulnerable to PTGR dysregulation than SCZ and BD ( ***Fig. 1D*** and ***Fig. 2A***), but also appeared to exhibit post-transcriptional up-regulation of mature mRNA levels, in contrast to the down-regulation observed in SCZ and BD ( ***Fig. 2A***). We further identified widespread genes whose expression was significantly post-transcriptionally altered in ASD, SCZ, and BD ( *n* = 1807, 1019, and 979 with a false discovery rate [FDR] < 0.05, respectively; ***Fig. 2B***), most of which were not shared across disorders. Nevertheless, transcriptome-wide comparison of ∆PTGR across three disorders revealed a significant SCZ/BD cross-disorder correlation ( ***Fig. 2C***), implying that SCZ and BD may share overlapping post-transcriptional etiology, while post-transcriptional dysregulation in ASD is more likely to be unique among the disorders ( ***Fig. 2C***).

To gain insight into the biological effects of PTGR dysregulation, we subsequently performed GO analysis on ASD-, SCZ-, and BD-associated DPRGs. The results showed that ASD-associated upregulated DPRGs were significantly enriched in the apoptotic process, while SCZ- and BD-associated upregulated DPRGs were significantly enriched in immune activation-related biological processes ( ***Fig. 2D***). Additionally, downregulated DPRGs were associated with neural development, neuron differentiation, and synaptic transmission in SCZ and BD; however, there was no enrichment of biological processes in ASD-associated downregulated DPRGs ( ***Fig. 2E***). Together, these results indicate that the disease specificity and complexity across the three disorders are partially attributed to PTGR dysregulation.

### Disease-specific RBPs modulated mature mRNA levels post-transcriptionally in psychiatric disorders

Because RBPs post-transcriptionally modulate mature mRNA levels *via* the adenylate-uridylate-rich elements (AREs) and regulate the expression of many genes at the post-transcriptional level ^[ 29] ^, we examined whether DPRGs were enriched with AREs. We observed ARE enrichment in DPRGs, but not in DEGs ( ***Fig. 1E***).

**Figure 3:**
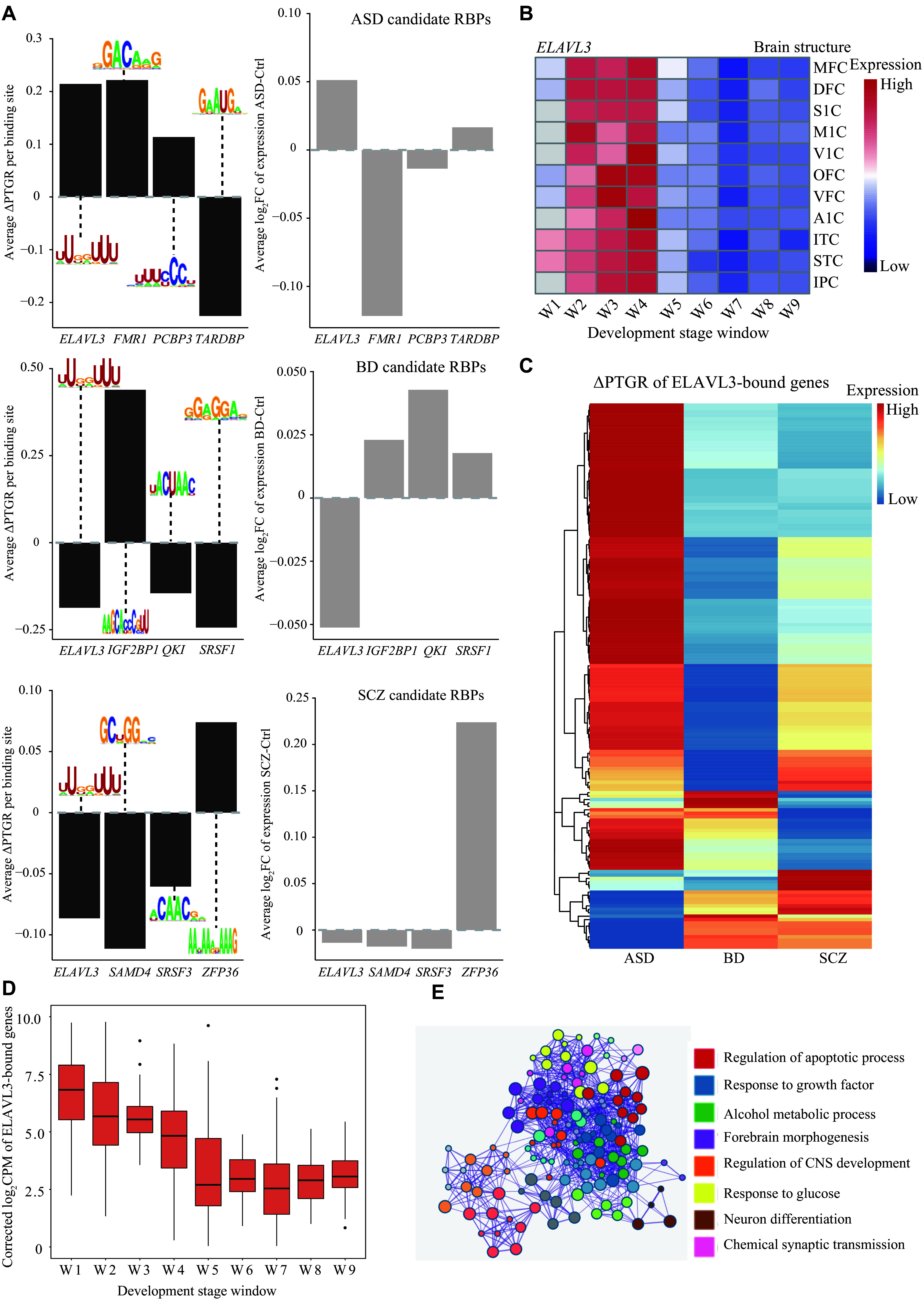
Disease-specific RNA binding-proteins (RBPs) that modulate RNA stability in psychiatric disorders. A: RBPs highly predictive of transcriptome-wide mRNA stability in different disorders are shown ( *P*-value < 0.05, *t*-test of regression coefficients). The bar plot (left) shows the predicted key RBPs and the corresponding RBP binding motifs. The y-axis within the left bar plot represents the average PTGR of predicted RBP-bound target genes in disease versus control. The bar plot (right) shows the average log _2_(fold change [FC]) of RBP expression in disease versus control. B: The normalized expression levels of *ELAVL3* in the developing brain across different developmental time points and brain regions. Data were obtained from the BrainSpan Atlas database. Red denotes high expression; blue denotes low expression. W: development stage window. W1: 8–9 post-conceptional weeks. W2: 12–13 post-conceptional weeks. W3: 16–17 post-conceptional weeks. W4: 19–21 post-conceptional weeks. W5: 24 post-conceptional weeks to 0.3 years. W6: 0.5–2 years. W7: 3–11 years. W8: 13–19 years. W9: 21–64 years. C: The heatmap shows the ∆PTGR of 159 predicted ELAVL3-bound genes across the three diseases. The ∆PTGR represents the changes of the PTGR in disease versus control. D: The box plot shows the average expression of 159 ELAVL3-bound genes at different developmental stages. The stage windows W1–9 correspond to the developmental time windows in ***Fig. 3B***. E: Enrichment analysis of ELAVL3-bound genes. Each color represents a type of biological process category. Abbreviations: PTGR, post-transcriptional gene regulation; MFC, rostral medial prefrontal cortex; DFC, dorsolateral prefrontal cortex; S1C, primary somatosensory cortex; M1C, primary motor cortex; V1C, primary visual cortex; OFC, orbital frontal cortex; VFC, ventrolateral prefrontal cortex; A1C, primary auditory cortex; ITC, inferolateral temporal cortex; STC, posterior superior temporal cortex; IPC, posteroventral parietal cortex.

This result supports the potential involvement of RBPs in modulating DPRGs across the three disorders. To predict the most responsible RBPs, we leveraged the sequence preference of known RBPs and examined the association between transcriptome-wide ∆PTGR and counts of binding sites of each RBP with multiple linear regression (see *Materials and methods* for more details). Consequently, we identified multiple RBPs that were highly predictive of transcriptome-wide alterations in post-transcriptional regulation in different disorders ( ***Fig. 3A***). Known ASD and SCZ risk RBPs, such as *FMR1* and *QKI* ^[ 30– 31] ^, were among the top candidates. Other promising candidates included neurodevelopment-associated *ELAVL3* ^[ 32]^ and *IGF2BP* family ^[ 33] ^, the neuroimmune-related gene *ZPF36*, and classical splicing factor *SRSF1*. More importantly, the effects of these RBPs on PTGR were consistent with their roles verified experimentally in the literature ^[ 34] ^. For instance, *FMR1* represses translation by reducing mRNA stability ^[ 35] ^. Our analysis predicted that transcriptome-wide targets of *FMR1* tended to increase in mRNA stability in ASD ( ***Fig. 3A*** top left), as expression of *FMR1* decreased in ASD ( ***Fig. 3A*** top right). Collectively, the present study provided an interesting list of risk RBPs worthy of further research attention.

### *ELAVL3* was responsible for the disruption of post-transcriptional regulation in all three disorders

Intriguingly, we found that *ELAVL3*, a neural-specific RNA-binding protein, was preferentially expressed during early brain development ( ***Fig. 3B***), among the top candidates for all three disorders, with opposite effects on PTGR between SCZ/BD and ASD ( ***Fig. 3A*** and ***3C***; ***Supplementary Fig. 1*** [available online]). *ELAVL3* was upregulated in ASD, and its targets were post-transcriptionally upregulated in ASD, while it was downregulated in SCZ/BD and its targets were post-transcriptionally downregulated in SCZ/BD ( ***Fig. 3A*** and ***3C***). Therefore, the presence of 3′ UTR binding sites for *ELAVL3* was significantly associated with post-transcriptional upregulation. We subsequently assessed the expression of 159 *ELAVL3*-bound genes and found that the average expression of these genes was also high in brain development ( ***Fig. 3D***), consolidating the important role of *ELAVL3* in early brain development. The *ELAVL3*-bound genes were significantly enriched in the core biological processes implicated in the three disorders mentioned above, such as neurodevelopment, regulation of the apoptotic process, and neuron differentiation ( ***Fig. 3E***). Overall, the present study pointed to an RBP whose malfunction may underlie the common pathogenesis shared by the three disorders.

### Knockdown of *ELAVL3* caused developmental defects of cerebral organoids

**Figure 4:**
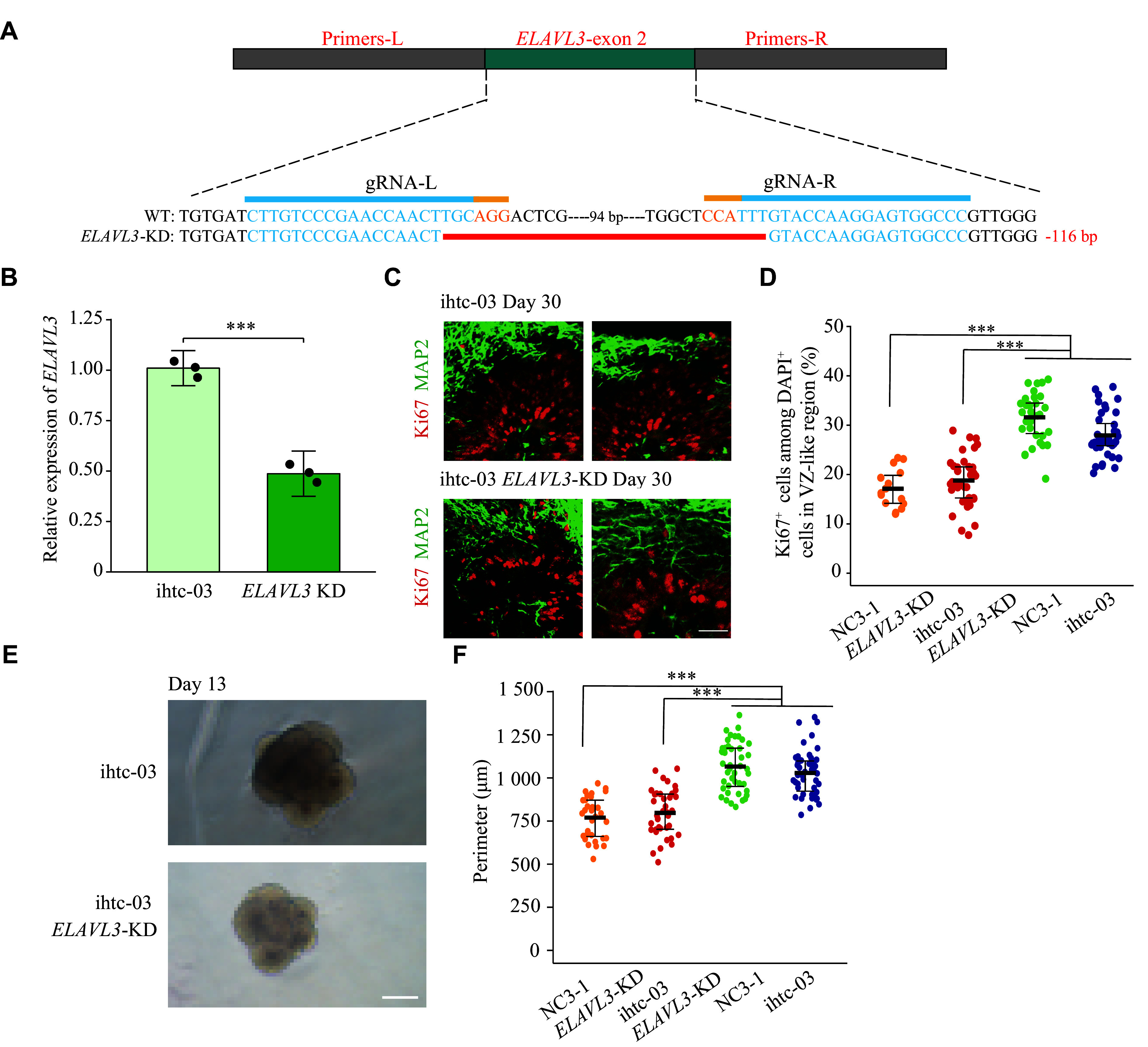
Phenotypic characterization of *ELAVL3*-knockdown (KD) cerebral organoids. A: A schematic diagram shows the generation of *ELAVL3*-knockdown organoids using CRISPR/Cas9 genome editing technology. Blue sequence: gRNA; yellow sequence: PAM sequences. B: Relative expression levels of *ELAVL3* in day 30 ihtc-03-derived control and *ELAVL3*-KD cerebral organoids, as assessed by qPCR. Data are reported as mean ± standard error of the mean (SEM; *n* = 3). ^***^ *P* < 0.001 by Student's *t*-test. C: Representative immunofluorescence images of Ki67-positive and MAP2-positive cells after 30 days of differentiation. Ki67: proliferating radial glial progenitor marker. MAP2: neuron marker. Top: control cerebral organoids at day 30. Bottom: *ELAVL3*-KD cerebral organoids at day 30. Scale bar: 20 μm. D: Quantification of the proportion of Ki67-positive cells in *ELAVL3*-KD and control organoids after 30 days of differentiation. *n* = 15–40 ventricular zone (VZ)-like regions in at least five organoids per cell line. Data are presented as mean ± SEM. ^***^ *P* < 0.001 by one-way ANOVA. E: Bright-field microscopic images of ihtc-03 differentiated cerebral organoids at day 13. Top: control cerebral organoids at day 13. Bottom: *ELAVL3*-KD cerebral organoids at day 13. Scale bar: 250 μm. F: Quantification of organoid perimeter on day 13. At least 38 EBs were analyzed for each cell line; *n* ≥ 3 independent experiments. Data are presented as mean ± SEM. ^***^ *P* < 0.001 by one-way ANOVA.

**Figure 5:**
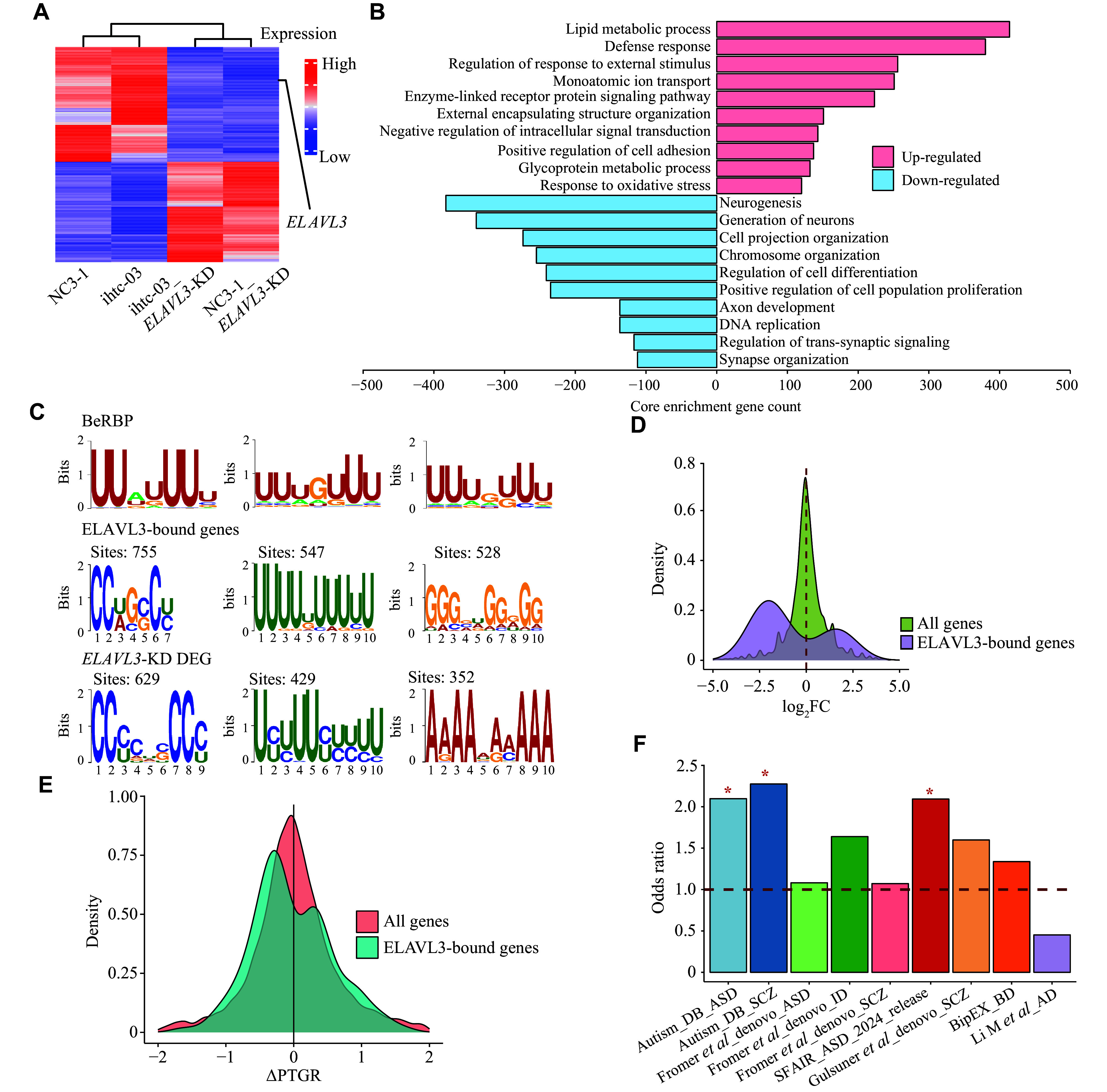
Transcriptional analyses in cerebral organoids derived from *ELAVL3*-KD and control iPSCs. A: Heatmap of significantly differentially expressed genes identified by comparing *ELAVL3*-KD cerebral organoids with control cerebral organoids using DESeq2. Highly expressed genes are colored red, and the lowly expressed genes are colored blue. B: Differentially expressed genes were enriched in GO terms based on the GSEA GO analysis, as ranked by the number of core enrichment gene counts. C: Top: classical RNA binding motif of ELAVL3 from the beRBP database. Middle: motif of the 3′ UTR sequence of 159 predicted ELAVL3-bound genes with significant ∆PTGR, enriched using MEME software. Bottom: motif of the 3′ UTR sequence of the significantly differentially expressed genes, *i.e.,* *P* < 0.05 and |log _2_(fold change [FC])| > 1, in *ELAVL3*-KD cerebral organoids, enriched using MEME software. D: The density plot shows the distribution of the expression changes of different gene sets in control and *ELAVL3*-KD cerebral organoids. The x-axis shows the log _2_FC of *ELAVL3*-KD versus control cerebral organoids. The purple color represents the ELAVL3-bound genes, and the green color represents all expressed genes in cerebral organoids. ELAVL3-bound genes were defined as in ***Fig. 3C***. E: The density plot shows the distribution of ∆PTGR of different gene sets in control and *ELAVL3*-KD cerebral organoids. The x-axis shows alterations in post-transcriptional regulation of mature mRNA levels ( *ELAVL3*-KD versus control) from RNA-Seq data. The blue color represents the ELAVL3-bound genes, and the pink color represents all expressed genes in cerebral organoids. ELAVL3-bound genes were defined as in ***Fig. 3C***. F: Enrichment of the significantly differentially expressed genes in *ELAVL3*-KD cerebral organoids across neuropsychiatric gene sets. The odds ratio was generated using Fisher's exact test. ^*^ *P* < 0.05. The autism_DB_ASD and autism_DB_SCZ gene sets were collated from the autism DB database. The Fromer *et al*_denovo_ASD, Fromer *et al*_denovo_ID, and Fromer *et al*_denovo_SCZ gene sets were collated from Fromer *et al* ^[ 25] ^. The SFAIR_ASD_2024_release gene set was collated from the SFAIR autism database. The Gulsuner S_denovo_SCZ gene set was collated from Gulsuner S *et al* ^[ 26] ^. The BipEX_BD gene set was collated from the BipEX database. The Li *et al*_AD gene set was collated from Li M *et al* ^[ 27] ^. Abbreviations: ∆PTGR, post-transcriptional gene regulatory change; ASD, autism spectrum disorders; SCZ, schizophrenia; ID, intellectual disability; BD, bipolar disorder; AD, alzheimer's disease.

To validate the post-transcriptional role of *ELAVL3* in neurodevelopment, we knocked it down in cerebral organoids, which are powerful 3D cellular model to study the early development of the human brain or neuropsychiatric disorders ( ***Fig. 4A***) ^[ 36] ^. *ELAVL3* downregulation was confirmed by qPCR ( ***Fig. 4B***). Intriguingly, the number of proliferating cells, marked by Ki67, was significantly decreased following *ELAVL3* knockdown ( ***Fig. 4C*** and ***4D***). Consequently, the size of the organoids was also significantly reduced ( ***Fig. 4E*** and ***4F***). Additionally, RNA-seq analysis identified 832 differentially expressed genes ( *ELAVL3*-KD-DEGs; ***Fig. 5A***), which were primarily involved in neurogenesis ( ***Fig. 5B***) and whose 3′ UTR sequences were enriched with the same binding motifs of *ELAVL3* as recorded in the beRBP database ( ***Fig. 5C***). The predicted 159 bound targets of *ELAVL3* tended to exhibit decreased expression and to be post-transcriptionally downregulated upon *ELAVL3* knockdown ( ***Fig. 5D*** and ***5E***). Moreover, the known ASD/BD/SCZ risk genes were overrepresented in *ELAVL3*-KD-DEGs ( ***Fig. 5F***), corroborating the link between common neuropsychiatric disorders and developmental defects due to abnormal expression of *ELAVL3*.

## Discussion

We determined the role of PTGR dysregulation in neuropsychiatric disorders, with a specific focus on its influence on gene expression. The present comprehensive study revealed for the first time that PTGR dysregulation is widespread and, unexpectedly, not negligible compared with TGR dysregulation, warranting further attention. The most significant finding is that, unlike the SCZ/BD brain, the ASD brain generally shows post-transcriptionally upregulated mature mRNA levels, implying a novel therapeutic approach to intervene in these diseases by manipulating the kinetics of RNA metabolism.

Our current understanding of the relationship between many diseases and PTGR is primarily restricted to miRNAs, which regulate the degradation and translation of hundreds of mRNAs by binding to their 3′ UTRs. Numerous well-characterized miRNAs, such as miR-132, miR-195, miR-188, and miR-137 ^[ 37] ^, have been associated with neuropsychiatric disorders, whereas the role of RBPs remains relatively unexplored. As far as we know, the human genome encodes more than 1500 RBPs, a number that is still expanding, yet only a small fraction has been functionally characterized ^[ 5] ^. Encouragingly, our study provides a list of RBPs of top priority for further exploration. Moreover, RBPs regulate the end products of expression more directly than transcription factors, making RBPs more popular targets for drug intervention ^[ 38] ^.

Different neuropsychiatric disorders are believed to share part of their pathogenesis, and some have proposed that there is a spectrum of neurodevelopmental disorders, on which ASD, BD, and SCZ lie at different ends ^[ 39] ^. From the post-transcriptional perspective, we showed that SCZ and BD might lie closer to each other but farther away from ASD on the spectrum. Notably, SCZ shared more risk loci with BD than with ASD ^[ 40] ^. Thus, the present study corroborated the shared pathogenesis between SCZ and BD from a different perspective. Several RBPs are involved in adult neurogenesis, including CPEB3, FXR2, FMRP, HuR, HuD, Lin28, Msi1, Sam68, Stau1, Smaug2, and SOX2, which regulate cell proliferation, differentiation, survival, and maturation as well as post-transcriptional gene expression ^[ 41] ^. Rare and common variants in *RBFOX1* are associated with a range of psychiatric disorders, including attention-deficit/hyperactivity disorder, ASD, BD, major depression, and SCZ ^[ 42– 43] ^. Mouse experiments showed that cytoplasmic *Rbfox1* increased the mRNA stability and translation of the target by binding to the 3ʹ UTR and participating in synaptic transmission and cortical development ^[ 44] ^. Our study showed that PTGR abnormalities in both SCZ and BD exhibited the inhibition of neuronal differentiation and synaptic transmission, while PTGR abnormalities in ASD demonstrated excessive activation of apoptosis. A pioneering study analyzed three key brain regions, including the hippocampus, cerebellum, and frontal cortex, in six autistic brains and six non-autistic brains from six- to 16-year-old deceased children, and observed a significant occurrence of endoplasmic reticulum stress, oxidative stress, and apoptosis in the autistic brain ^[ 45] ^. One study has shown that amyloid-beta peptides may trigger acquired microcephaly in ASD patients through pathways such as disrupting neurogenesis and promoting cellular apoptosis ^[ 46] ^. The biological effects of PTGR dysregulation in ASD observed in our study are consistent with the increased apoptosis in the autistic brain. These findings suggest the potential biological consequences of excessive activation of apoptosis due to dysregulation of PTGR in autism, providing clues for future research. Additionally, the abnormality of brain neural circuits caused by dysregulation of synaptic organization, differentiation, and transmission is a key molecular mechanism of neurodevelopmental, neuropsychiatric, and neurodegenerative diseases ^[ 47] ^. The synaptic pathophysiological changes are consistent with the pathways affected by PTGR abnormalities in SCZ and BD. This implies that the synaptic and neuronal differentiation abnormalities caused by PTGR dysregulation in SCZ and BD may influence connectivity in brain circuits, but this requires further exploration. Furthermore, our findings reveal distinct sets of RBPs responsible for different disorders, providing support for the goal of not relying on descriptive syndromes but on a nosology guided by susceptible genes in psychiatry.

ELAVL3 appeared to be involved in the pathogenesis of all three disorders, although its alteration in expression and PTGR was disorder-specific. ELAVL3 belongs to the ELAVL family of RNA-binding proteins, all members of which function in a spatiotemporal manner to regulate brain development ^[ 48] ^. With regard to the molecular mechanism, a previous study showed that Elavl3 influences post-transcriptional RNA modification by lengthening the 3′ UTR, consistent with our prediction ^[ 49] ^. Although the Simons Foundation Autism Research Initiative (SFARI) lists *ELAVL3* as a risk gene underlying ASD ^[ 50] ^, research on *ELAVL3* has focused on putative roles in amyotrophic lateral sclerosis, supported by animal models showing ataxia in the elderly and *ELAVL3*'s expression in Purkinje neurons ^[ 51] ^. However, the validation in cerebral organoids suggested that *ELAVL3* might have a species-specific role. *Elavl3* ^−/−^ null mice did not exhibit obvious brain defects ^[ 32] ^, while there was an arrest in neurodevelopment following *ELAVL3* knockdown in organoids. *ELAVL3*-knockdown cerebral organoids in our study showed diminished proliferation and reduced size. Additionally, iPSC-derived cerebral organoids from monozygotic twins with discordant psychosis (schizoaffective disorder, bipolar type) exhibited reduced cell proliferation following diminished Wnt signaling at the early stage of neurodevelopment ^[ 52] ^. Autism-like mice derived from *Gigyf1* haploinsufficiency in the developing mouse brain have fewer neurons in the upper cortex, accompanied by decreased proliferation and increased differentiation of neural progenitor cells ^[ 53] ^. These findings are consistent with our results and simultaneously support the link between diminished cell proliferation and psychiatric disorders. Sawada *et al* ^[ 52]^ speculated that decreased proliferation during neurodevelopment might affect the brain volume of psychosis-affected individuals before the onset of illness. Another study on SCZ high-risk populations consistently indicates that compared with the control group, the cortical gray matter volume of the diseased population is smaller, suggesting that brain volume is already reduced at the onset of illness, with further reduction in gray matter volume after onset ^[ 54] ^. These results suggest that decreased cell proliferation may be associated with reduced brain volume in patient populations, but further exploration is needed. Note that post-transcriptional changes upon *ELAVL3*-KD are not well correlated with the changes in mRNA half-life determined by transcriptional inhibition with actinomycin D (not shown), suggesting that the primary role of *ELAVL3* is unlikely to protect bound targets from degradation.

Future research is warranted to better understand the function of *ELAVL3* in human brain development, particularly in cortical development, and the malfunction of *ELAVL3* underlying neuropsychiatric diseases, which may lead us to the core pathogenesis of these diseases.

## SUPPLEMENTARY DATA

Supplementary data to this article can be found online.
