# Supplementary material for: Post-transcriptional dysregulation in autism, schizophrenia, and bipolar disorder
Source: J Biomed Res. 2024 May 29;39(4):325–39. doi: 10.7555/JBR.38.20240114 (PMC12336410; doi:10.7555/JBR.38.20240114)
Supplement: Supplementary file 1 — Supplementary data to this article can be found online. [file jbr-39-4-325-Supplementary.pdf]

Available online at www.jbr-pub.org.cn

Open Access at PubMed Central

Journal of Biomedical Research, 2025 39(4): 1–1

Supplementary Data

JBR

Post-transcriptional dysregulation in autism, schizophrenia, and bipolar disorder

Yuanyuan Wang[1,2,△], Yitong Yan[2,△], Bin Zhou[3,✉], Mingyan Lin[2,✉]

[1]*State Key Laboratory of Reproductive Medicine and Offspring Health, Nanjing Medical University, Nanjing, Jiangsu 211166, China;*
[2]*Department of Neurobiology, School of Basic Medical Sciences, Nanjing Medical University, Nanjing, Jiangsu 211166, China;*
[3]*Department of Cardiology, the First Affiliated Hospital of Nanjing Medical University, Nanjing, Jiangsu 210029, China.*

Data in ***Supplementary Table 1*** (available online) include the top 100 differentially post-transcriptionally regulated genes ranked by mRNA stability changes across schizophrenia, bipolar disorder, and autism spectrum disorder. "Up" indicates that RNA stability tends to increase in disease. "Down" indicates that RNA stability tends to decrease in disease.

Data in ***Supplementary Table 2*** (available online) include the bulk RNA-Seq differential expression analysis results between *ELAVL3* knockdown organoids and control organoids. $\log_2$(fold change) > 0 indicates genes that are upregulated in *ELAVL3* knockdown organoids. $\log_2$(fold change) < 0 indicates genes that are downregulated in *ELAVL3* knockdown organoids.

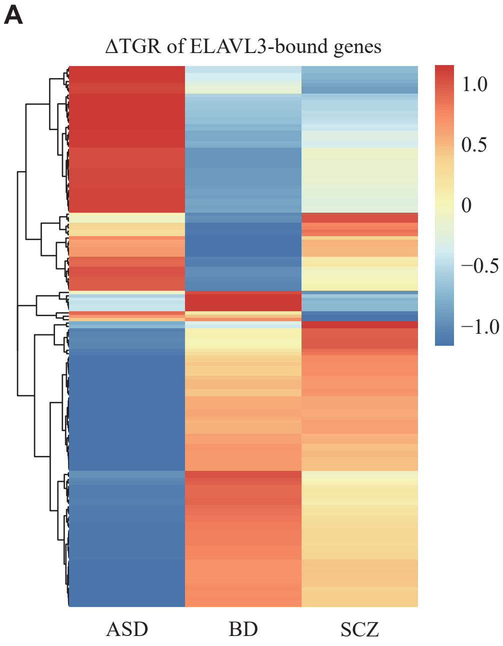

Supplementary Fig. 1 **Effects of *ELAVL3* on transcriptional regulation across three neuropsychiatric disorders.** The heatmap shows transcriptional gene regulation (TGR) changes of 159 predicted *ELAVL3*-bound genes across the three neuropsychiatric disorders. ELAVL3-bound genes were defined as in ***Fig. 3C***. Abbreviations: ΔTGR, transcriptional regulation changes; ASD, autism spectrum disorder; BD, bipolar disorder; SCZ, schizophrenia.

[△]These authors contributed equally to this work.

[✉]Corresponding authors: Mingyan Lin, Department of Neurobiology, School of Basic Medical Sciences, Nanjing Medical University, 101 Longmian Avenue, Jiangning District, Nanjing, Jiangsu 211166, China. E-mail: linmingyan@njmu.edu.cn; Bin Zhou, Department of Cardiology, the First Affiliated Hospital of Nanjing Medical University, 300 Guangzhou Road, Nanjing, Jiangsu 210029, China. E-mail: bin_zhou@yahoo.com.

Received: 18 April 2024; Revised: 20 May 2024; Accepted: 23 May 2024; Published online: 29 May 2024

CLC number: R749, Document code: A

The authors reported no conflict of interests.

This is an open access article under the Creative Commons Attribution (CC BY 4.0) license, which permits others to distribute, remix, adapt and build upon this work, for commercial use, provided the original work is properly cited.

© 2025 by Journal of Biomedical Research.

https://doi.org/10.7555/JBR.38.20240114
